# Cognitive therapy for post-traumatic stress disorder following critical illness and intensive care unit admission

**DOI:** 10.1017/S1754470X2000015X

**Published:** 2020-04-29

**Authors:** Hannah Murray, Nick Grey, Jennifer Wild, Emma Warnock-Parkes, Alice Kerr, David M. Clark, Anke Ehlers

**Affiliations:** 1 Department of Experimental Psychology, University of Oxford, Oxford, UK; 2 Oxford Health NHS Foundation Trust, Oxford, UK; 3 Sussex Partnership NHS Foundation Trust, UK; 4 University of Sussex, UK; 5 King’s College London, London, UK

**Keywords:** COVID-19, critical care, hallucinations, ICU, PTSD, trauma

## Abstract

Around a quarter of patients treated in intensive care units (ICUs) will develop symptoms of post-traumatic stress disorder (PTSD). Given the dramatic increase in ICU admissions during the COVID-19 pandemic, clinicians are likely to see a rise in post-ICU PTSD cases in the coming months. Post-ICU PTSD can present various challenges to clinicians, and no clinical guidelines have been published for delivering trauma-focused cognitive behavioural therapy with this population. In this article, we describe how to use cognitive therapy for PTSD (CT-PTSD), a first line treatment for PTSD recommended by the National Institute for Health and Care Excellence. Using clinical case examples, we outline the key techniques involved in CT-PTSD, and describe their application to treating patients with PTSD following ICU.

**Key learning aims:**

1. To recognise PTSD following admissions to intensive care units (ICUs).
2. To understand how the ICU experience can lead to PTSD development.
3. To understand how Ehlers and Clark’s (2000) cognitive model of PTSD can be applied to post-ICU PTSD.
4. To be able to apply cognitive therapy for PTSD to patients with post-ICU PTSD.

## Introduction

Patients are admitted into intensive care units (ICU) when they need life-saving medical treatment. Medical advances have led to higher survival rates in ICU than ever before, but many patients experience psychological difficulties in the weeks and months after discharge, particularly post-traumatic stress disorder (PTSD), depression and anxiety. Indeed, a recent meta-analysis found self-reported PTSD symptoms in 24% of ICU patients between 1 and 6 months after discharge, and 22% at 7 months (Parker *et al.*, 2015).

Various risk factors associated with admissions to ICU lead to higher rates of PTSD than other medical settings. Besides being critically unwell and fearing they may die, patients are exposed to the ICU environment with constant noise, light, frequent medical checks, pain, sleep disruption, partial consciousness and high levels of sedating medications. They are often unable to communicate or to move. These factors place patients at risk of delirium, an acutely confused state of mind that can be accompanied by hallucinations and delusions. The patient’s poor physiological state and the ICU environment also affect memory processing, further raising the risk of PTSD development.

The COVID-19 pandemic has led to a rapid increase in ICU admissions. One of the many aftershocks of the pandemic is likely to be a rise in patients presenting with PTSD related to their experiences of illness and medical treatment. As yet, data are not available to estimate rates of PTSD in this population or on specific PTSD risk factors such as the use of sedating medications. However, certain aspects of treatment during the pandemic may place patients at risk of developing post-ICU PTSD. Staff need to wear personal protective equipment (PPE), including masks which hamper communication and can make them look frightening to patients experiencing delirium. Visitors are not allowed unless the patient is dying, removing the opportunity for reassurance and support from a loved one, and staff will be exceptionally busy. In usual circumstances, approximately 13% of patients admitted to ICU die [Intensive Care National Audit and Research Centre (ICNARC), 2019], but early figures regarding COVID-19 cases indicate that mortality may be higher for this population, at over 50% at the time of writing (ICNARC, 2020), meaning that patients may be aware of others dying around them, and of their own high risk of death. Finally, critically ill COVID-19 patients experience hypoxia, which is a risk factor in delirium (Tilouche *et al.*, 2018). Some aspects of being unwell with COVID-19 may be protective of PTSD. For example, many patients in ICU have no memory of arriving there, as their admission was due to a sudden accident, illness or following an operation. COVID-19 patients have typically become increasingly unwell over a period of time. They may therefore have greater awareness of their situation, and more access to factual memories, which has been found to be protective of PTSD (Jones *et al.*, 2001).

The National Institute for Health and Care Excellence guidelines on rehabilitation after critical illness (NICE, 2009) recommend psychological follow-up for patients after discharge from ICU, including screening for PTSD, and psychological therapy as required. However, at the time of writing, follow-up clinics were routinely available in only some UK hospitals, and are likely to be over-stretched during the COVID-19 pandemic. Furthermore, patients with PTSD symptoms may be unable or unwilling to attend hospital follow-up clinics, due to ongoing physical problems and the distress associated with reminders of their experiences in hospital.

When it comes to treatment, there is little published clinical guidance available for psychological therapists working with patients with post-ICU PTSD. Clinical interventions in the literature have tended to focus on prevention of PTSD, including adjusting sedation and medication use (Kress *et al.*, 2003), the use of patient diaries (Ullman *et al.*, 2015), and early intervention programmes (e.g. Wade *et al.*, 2019). Treating post-ICU PTSD may present various challenges to clinicians, such as: the role of patchy and highly disjointed memories that may include hallucinations; ongoing physical problems; avoidance of healthcare-related triggers; and a distrust and fear of medical professionals.

In this article, we aim to describe how to apply cognitive therapy for PTSD (CT-PTSD), a first-line psychological treatment for PTSD, to meet the needs of post-ICU patients. Although we will focus predominantly on PTSD following treatment in ICU, much of the content is relevant to surviving critical illness in general, as well to medical traumas in other contexts.

## PTSD after ICU

*Traumatic events in ICU may include:*

- Experiences where the patient believed they were about to die.
- Moments when the patient learnt bad news, such as realising they had developed COVID-19 and related problems.
- ‘Flashforwards’ or images of a feared future event (such as imagining their own funeral).
- Invasive (and sometimes painful) medical procedures.
- Seeing, hearing or learning about other patients dying.
- Perceived mistreatment, such as experiencing pain and believing staff aren’t helping.
- Witnessing other patients behave in a distressing manner.
- Hallucinations caused by delirium.
- A combination of the above. For example, patients may have memories of a medical procedure, which they believed at the time was the nurse trying to kill them.

To meet criteria for PTSD, according to the *Diagnostic and Statistical Manual of Mental Disorders* (5th edn, DSM-5; American Psychiatric Association, 2013), as well as experiencing a Criterion A event (witnessing or experiencing actual or threatened death or serious injury), such as those listed above, the patient must also have symptoms from each of the following categories:

- *Re-experiencing the events in the form of intrusive memories, flashbacks, nightmares and/or physical and emotional reactivity to reminders* (*Criterion B*). With respect to ICU trauma, patients may report factual memories, delusional or hallucinated memories, or a mixture of the two (Colville *et al.*, 2008; Wade *et al.*, 2015). In some cases, they will report confusion about what was real. Re-experiencing does not always involve conscious recall of traumatic events. It can also involve re-experiencing an intense emotion (fear, sadness, despair) or physical reaction (pain, shortness of breath, immobility) from the trauma without simultaneously recalling the event itself. This is what Ehlers and Clark (2000) called ‘affect without recollection’. Triggers for re-experiencing include obvious reminders of the trauma (such as the mention of ICU, being asked about your illness, etc.) but also often include sensory elements of the admission that are ubiquitous in everyday life, such as beeping noises, smells of disinfectants, the sound of laboured breathing, the colour of PPE gowns, the clear plastic used in staff PPE visors, and physical sensations such as pain, difficulty breathing, discomfort when swallowing, lying down and nausea. When these sensory elements are part of everyday objects (the colour of clothes, the material used in household containers, the breathing of a newborn baby), patients may not be aware what has triggered their memories or affect and feel their emotions are out of control.
- *Avoidance of thoughts, feelings and reminders of the experience* (*Criterion C*). With respect to post-ICU PTSD, types of behavioural avoidance may include skipping medical appointments, avoiding looking at or touching parts of their own body, activities that bring on similar body sensations (such as getting out of breath) and turning off TV programmes or films with medical themes.
- *Negative alterations in cognitions and mood* (*Criterion D*). For PTSD following ICU, cognitions may relate to perceived negative, permanent changes to the self, one’s body or life in general, beliefs about personal vulnerability, and distrust of others, especially if patients believed they were mistreated in hospital. Related emotions may include hopelessness, sadness, shame and anger.
- *Hyperarousal symptoms* (*Criterion E*). Particularly common symptoms post-ICU are hypervigilance to internal states, such as sensations or symptoms which may indicate possible illness. Sleep is often poor, as lying in bed may trigger memories.

The symptoms need to have lasted for at least a month (Criterion E), cause significant distress and/or interference to important areas of functioning (Criterion F) and not be attributable to a substance or medical condition (Criterion G). The PTSD criteria in ICD-11 (World Health Organisation, 2018) focus more narrowly on re-experiencing in the form of flashbacks and nightmares, avoidance, hypervigilance and startle responses.

PTSD symptoms may develop immediately after ICU, but we have also observed cases of delayed onset. Patients often report that initially their attention was focused on their physical recovery, and it was only later that their psychological symptoms emerged or assumed priority.

## A cognitive model of PTSD

Ehlers and Clark’s (2000) cognitive model of PTSD suggests that the core experience of PTSD is a sense of serious current threat even though the trauma is in the past. This perceived current threat can be physical (‘I’m going to die’; ‘The world is a dangerous place’) and/or psychological (‘I’m weak’; ‘I’m all alone’).

The sense of threat is maintained by three processes. The first relates to meanings that arise from the way an individual has appraised the traumatic event or its aftermath. For example, if patients now see themselves and their loved ones as more vulnerable to critical illness or death and mistrust medical professionals, this will create an ongoing sense of threat.

The second concerns the nature of the trauma memory. The model suggests that because the trauma is processed in a predominantly sensory way (as a stream of sensory impressions) and sometimes also as unreal/not happening to the self, the worst moments of the trauma are poorly elaborated and disjointed from other autobiographical information in memory. This accounts for the ‘here and now’ quality of PTSD memories; when they are recalled, people may be unable to access other information that could correct impressions or negative beliefs they had at the time. In other words, the memory for these moments has not been updated with what the individual knows now, such as that they survived and that the meanings of the worst moments may be inaccurate (e.g. that the medical staff did not torture them, but in fact did the procedures to save their life). These types of memories are easily triggered by sensory cues that are similar to those encountered at the time of the trauma.

The third process maintaining the sense of current threat is the cognitive and behavioural coping strategies that the patient uses to attempt to reduce their sense of threat. These strategies can inadvertently increase symptoms (e.g. memory suppression or substance use) or the sense of threat (e.g. hypervigilance to danger). Importantly, avoidance, safety behaviours and rumination prevent change (re-appraisal) of traumatic meanings or in the nature of the trauma memory, which remains in its poorly elaborated state.

## Psychological risk factors for post-ICU PTSD

Most research into risk factors for post-ICU PTSD has focused on patient demographics and medical variables. For example, some types of medication (including benzodiazepines and opiates) are particularly associated with later PTSD development, probably due to their role in delirium (e.g. Bienvenu *et al.*, 2013; Girard *et al.*, 2007). The psychological variables which have been studied give us some insight into the processes leading to post-ICU PTSD and align with the cognitive model. For example, studies have shown that the number of traumatic medical events which occur in ICU (such as difficulty breathing) is not predictive of PTSD, but the experience of fear and stress is (Wade *et al.*, 2013). This fits with the cognitive model of PTSD, which suggests that it is the nature of the appraisal made about traumatic events as threatening which leads to PTSD.

Second, it seems likely that the effects of the ICU environment on memory processing are profound; the combination of strong medication, interrupted sleep and partial and varying levels of consciousness can lead to highly disorganised memories dominated by sensory information and not easily integrated with other autobiographical memories and information, making them readily triggered. Research shows that patients who experienced delusional memories (Kiekkas *et al.*, 2010), were less aware of their surroundings (Elliott *et al.*, 2016; Rattray *et al.*, 2010) and had a low sense of coherence (Valsø *et al.*, 2020) were more likely to develop PTSD after ICU. Studies that have shown the presence of factual memories is protective from PTSD (Jones *et al.*, 2001) suggest that, even if factual memories are unpleasant, they help patients to better contextualise their experiences, and add coherence and meaning to what is happening.

Lastly, research shows that patients with pre-existing psychological problems are more likely to experience delusions in ICU (Jones *et al.*, 2001) and to develop PTSD afterwards (Davydow *et al.*, 2008; Morrissey and Collier, 2016; Wade *et al.*, 2012). Prior trauma exposure has been linked to increased risk of post-ICU PTSD (Paparrigopoulos *et al.*, 2014) and themes from previous trauma often arise in hallucinations. Ehlers and Clark’s cognitive model of PTSD proposes that previous beliefs and experiences affect the nature of the trauma memory and the appraisals formed. For example, someone with a pre-existing anxiety disorder or trauma history is more likely to interpret events in ICU as threatening.

**Figure 1.**
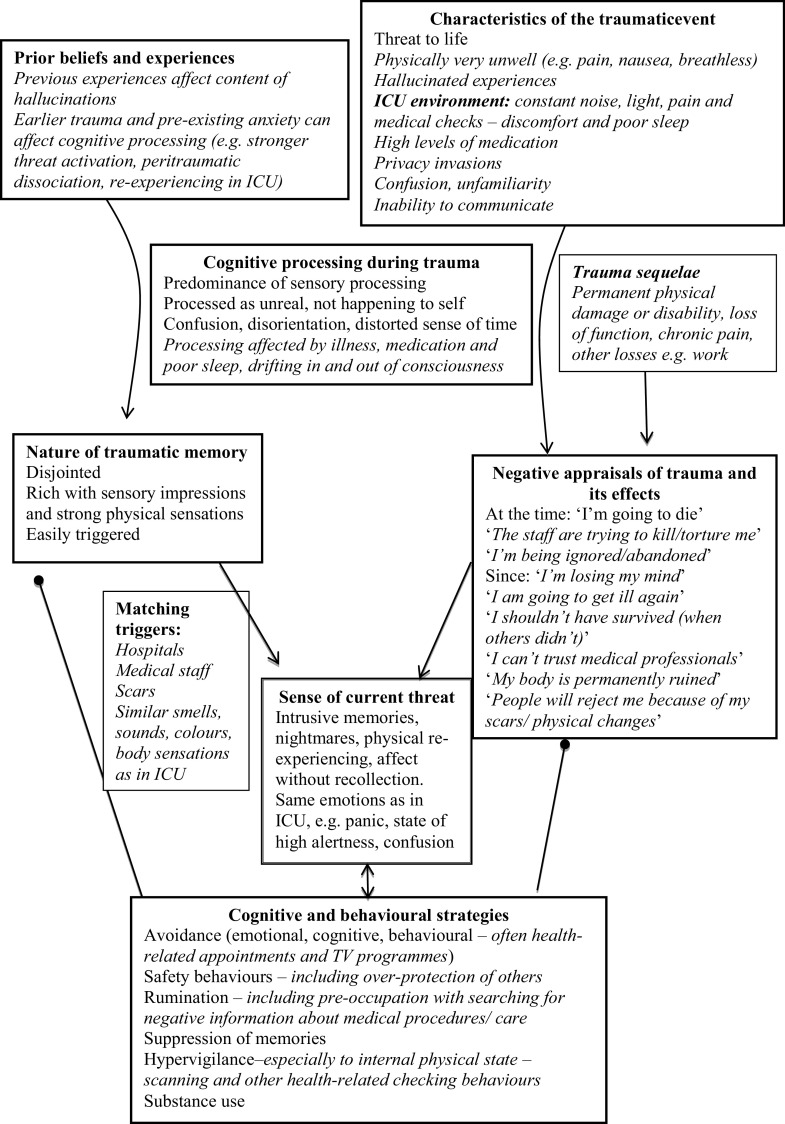
Ehlers and Clark’s (2000) cognitive model of PTSD, applied to post-ICU PTSD.

Figure 1 shows the cognitive model for PTSD adapted to illustrate typical features of post-ICU PTSD (in italics).

## Cognitive therapy for PTSD

Ehlers and Clark’s (2000) cognitive model of PTSD forms the basis of cognitive therapy for PTSD (CT-PTSD), a trauma-focused cognitive behavioural therapy recommended by NICE guidelines (NICE, 2018). CT-PTSD has demonstrated efficacy in randomised controlled trials (Ehlers *et al.*, 2003, 2005, 2014), and in routine clinical practice (Ehlers *et al.*, 2013). Treatment usually consists of up to 12 weekly sessions of 90 minutes, with up to three monthly follow-up sessions.

In line with the model, the aims of CT-PTSD are as follows:

- To modify threatening appraisals (personal meanings) of the trauma and its sequelae.
- To reduce re-experiencing by elaboration of the trauma memories and by breaking the link between everyday stimuli and trauma memories (then *vs* now trigger discrimination training).
- To reduce cognitive strategies and behaviours that maintain a sense of current threat.

For further information on how to conduct CT-PTSD, including training videos, questionnaires to guide treatment, guidelines for conducting treatment remotely, and post-ICU PTSD information leaflets, go to: www.oxcadatresources.com. These training materials assume existing training and competence in CBT.

## Using CT-PTSD for treatment of post-ICU PTSD

The core treatment strategies of CT-PTSD can all be used with patients with post-ICU PTSD. The following suggestions are examples of how the core techniques can be applied with these patients. A summary table (Table 1) is provided.

**Table 1.** CT-PTSD treatment strategies with ICU-PTSD applications

CT-PTSD treatment technique	ICU-PTSD application
Psychoeducation and normalisation	Include information about ICU-PTSD and delirium (if experienced), include information about physical reexperiencing and affect without recollection, and that memory gaps are common due to illness and medication
Individualised case formulation	Include the impact of the ICU environment, and pre-existing experiences (if relevant)
Reclaiming your life	Reframe as ‘rebuilding your life’ for people with significant losses or physical changes, include in each session. Pace according to pain and/or disability
Memory-focused techniques	
Updating the trauma memory	Use timelines to provide an overview of ICU stay and/or a written narrative, even though trauma memories are likely to be very disorganised or may contain hallucinations Gaps are acknowledged (‘the next thing I remember is…’) To access meanings of particular hotspots, reliving of these moments is helpful Updates may include better than expected outcome (‘I did not die’), reasons for interventions, intention of staff to help, reasons for being alone, distorted sense of time due to illness, sleep disturbance and lack of day light (see Table 2 for examples) Include updates to make sense of delusions/hallucinations Consult medical notes and/or experts to generate possible updates
Trigger discrimination	Detective work identifying audio/visual/olfactory stimuli or bodily sensations that were present in ICU but are also ubiquitous in the post-ICU environment and act as triggers for trauma re-experiencing Then vs Now discrimination may involve behaving during memory elicitation in ways that were not possible during traumatic moments in ICU (i.e. standing up, moving around)
Site visits	Arrange to visit the ICU by contacting the ward where possible Prioritise visits if trauma memories include delusions or hallucinations Use virtual site visits and video tours of ICUs to prepare for site visits or if you cannot return in person
Working on meanings of the trauma and its aftermath	Common themes include: Beliefs about losing one’s mind – use psychoeducation, research, surveys and behavioural experiments Belief about permanent physical change – acknowledge and mourn losses, increase focus on what has not changed; photo and video feedback and surveys to address distortions about severity of changes in appearance/function Belief about permanent psychological change – identify and challenge loss of confidence in one’s own abilities (‘I can no longer be trusted to look after my family’) Health anxiety and beliefs about vulnerability to illness – use guided discovery and medical advice to calculate accurate risk probabilities; drop scanning of body sensations/signs of illness; behavioural experiments Survivor guilt – address beliefs about perceived responsibility and inequity, consider alternative meanings, use surveys Beliefs about mistreatment and mistrust of healthcare professionals – listen and empathise, address misappraisals, facilitate communication with hospital, reviews costs and benefits of holding on to anger, reduce rumination Address generalised appraisals re: trust by reviewing evidence, surveys and research
Address maintaining behaviours/cognitive strategies	Increase awareness of strategies, e.g. internal scanning, over-protectiveness, rumination, substance use and their role in maintaining PTSD Use behavioural experiments to test effects of strategies, including experiments in reducing and dropping strategies and experiments to modify appraisals that motivate the behaviours

### Psychoeducation and normalisation

In the early stages of CT-PTSD, we use psychoeducation to help patients understand PTSD and to normalise their symptoms. An example leaflet, and a specific version for post-ICU PTSD, can be found on the OxCADAT resources website. With post-ICU PTSD, it is also helpful to give normalising information about ICU traumas. This is important because patients may have beliefs about their experiences such as ‘I’m losing my mind’ (especially in the case of delirium) and ‘I should be over this by now’.

Psychoeducation could include:

- That PTSD is common after ICU (20–30% of patients experience PTSD symptoms).
- A nurse or other specialist giving audio or written feedback on why some medical procedures are carried out in ICU.
- That delirium in ICU is extremely common (60–80% of patients).
- Why delirium occurs in ICU (most likely a combination of strong medications, sleep deprivation and/or hypoxia).
- That hallucinations and delusions in ICU are not signs of mental illness. They tend to only occur in that specific environment and do not lead to the development of a psychotic illness.
- Memories of experiences in ICU are usually muddled, without a clear sense of time, and can come back in various forms: memories of certain moments, feelings in the body, or sudden feelings such as panic. All of these are signs that a memory has been triggered.

Normalising information can also be found by reading first-person accounts of ICU experiences (e.g. by journalist David Aaronovitch: https://bbc.in/2WuueQy) or accessing online resources or support groups for ICU survivors (e.g. ICU Steps: www.icusteps.org).

### Individualised case formulation

Another early task in CT-PTSD is developing an individualised case formulation with the patient. This is not as detailed as in Fig. 1, but includes a basic description of the main processes maintaining their PTSD (i.e. the sources of the sense of current threat and any problematic cognitive or behavioural strategies; see the OxCADAT resources website for a video on developing formulations). With post-ICU PTSD, we also discuss the impact of the ICU context on memory processing, emphasising that there is little wonder that their trauma memories were difficult to make sense of, or to ‘put away’ in the memory system, given the intense physiological and environmental experience of ICU.

Learning a little about the patient’s pre-hospital experiences and beliefs may also reveal links to the content of delusions and hallucinations. It can be helpful to draw these links with the patient to understand their delirium experiences better.

*Shannon had been sexually abused by her uncle in childhood. In ICU, she experienced intensely distressing hallucinations of being sexually assaulted by staff. On discussion with her therapist, Shannon realised that the nature of her hallucinations may have been influenced by her previous trauma. It was likely that staff sometimes touched Shannon intimately for medical reasons, such as when washing her or adjusting her catheter. Shannon knew that she had been in and out of consciousness, and frequently confused while in ICU, and realised that she may have misinterpreted these procedures as sexual assaults, something she was understandably particularly fearful of*.

### Reclaiming/rebuilding your life

Reclaiming previously valued and enjoyed activities or equivalents after a trauma is an important part of CT-PTSD, which starts in session 1 and is reviewed every session. Following a critical illness, some patients have significant physical changes, including pain, disability, scarring, sexual dysfunction and ongoing symptoms of chronic health conditions. The longer-term physical consequences of COVID-19 are not yet known, but may include fatigue and respiratory problems. There may have been other significant changes to their life, like being unable to work, financial problems, or lifestyle changes. These obstacles may make it difficult to fully reclaim their life as it was before.

Reclaiming your life will emphasise ‘rebuilding’ your life after trauma. Work creatively and collaboratively with your patient to identify what is possible to reclaim, and how to replicate what was important to the patient about previously meaningful activities in other ways. You may need to pace work with your patient to accommodate pain or disability. What is possible will also vary over time, depending how soon after ICU a patient is seen, and the stage of their physical recovery. Plans for future rebuilding your life tasks can be made to match further physical improvements.

Gwilym had been a senior police officer before his illness and treatment in an ICU. He was left with significant pain and partial paralysis, meaning he had to give up both his career in the police and some of his hobbies. Gwilym and his therapist worked together on how to rebuild his life. They started by listing the things Gwilym had enjoyed which he could still do, including watching sport on the TV, going to the pub with friends, and sitting in the garden with his wife, and made a plan to reintroduce these activities. They then discussed what Gwilym had enjoyed about his job, and his other hobbies. Gwilym identified the feeling of doing something positive for his community, the camaraderie with his team and being physically active. They discussed other opportunities to achieve these aims, in ways which were physically possible for Gwilym. He decided to start volunteering for a local charity, producing their newsletter, which he could do from home. He also used his expertise to set up a Neighbourhood Watch in his local area, which had the added benefit of getting to know his neighbours better. He made an effort to stay in touch with colleagues, and other friends, and made plans to meet them. Gwilym also began to gradually increase his physical fitness, using a plan given to him by the hospital physiotherapists.

### Memory-focused techniques

#### Updating trauma memories

The first step to updating trauma memories in CT-PTSD involves accessing problematic meanings through imaginal reliving or creating a written narrative of the experience. As stays in ICU can be prolonged and the patient may have only been conscious for part of it, reliving or writing about it in detail from start to finish will be difficult. Patients often lose sense of time in ICU, especially if they experience delirium. Where possible, it can be helpful to construct a rough timeline of their stay in ICU, using information from medical records, ICU diaries (which are used in some units) and recollections from family or friends who, even if they were not allowed to visit, hopefully received regular updates from the ward. This provides an overview of the stay, and trauma memories that are currently being re-experienced can be identified and marked on the timeline.

Writing a narrative of what the patient remembers is also useful to provide an overview of their ICU experience and to identify hotspots (moments of peak emotional distress during the trauma memory). Patients and therapist can discuss different possibilities for confusing moments while writing together and constructing a plausible sequence of events. Confusion and gaps are acknowledged in the narrative (‘the next thing I remember is…’). The therapist monitors reactions such as signs of dissociation, feeling faint, confused, nauseous or pain to gauge the pace of the engagement with the trauma memories and can remind the patient of the present as needed or use applied tension if the patient starts to feel faint.

The events around the particular memories that are re-experienced or cause the greatest distress during the narrative writing or constructing the timeline (hotspots) can then be explored in more depth through imaginal reliving to fully access their meanings. Updating information is then identified and brought into the trauma memory as early as possible. The updates are written into the narrative account in a different colour or font. The patient or therapist reads the hotspot and update out loud, while the patient holds both in mind. In imaginal reliving, the therapist prompts the patient to say the update out loud when they reach the hotspot in the memory and to hold an image of the update in mind. It can also be useful to use actions or images to remind themselves of the update. For example, incompatible actions or sensations such as moving about or touching their healed body can show that the danger has passed, and they have recovered. Looking at photos of recent events with loved ones can update moments where patients may have feared they would die without saying goodbye to their loved ones. Images such as the body healing after illness to strengthen the update ‘the illness has passed, my body is healthier now’. Ratings of memory ‘nowness’ (the extent to which the event feels as if it is happening again) and distress are collected before and after updating to check that the procedure has been successful. Video examples of this important technique are available on the OxCADAT resources website.

The meanings of hotspots are individual to each person, and are identified through careful questioning. Table 2 lists some common hotspot meanings from several different post-ICU PTSD patients, together with possible updates and ways of demonstrating the updated meanings.

**Table 2.** Example hotspots and updates for ICU trauma memories

	Example update
I’m going to die, I’m never going to see my family again	I didn’t die, I’m alive. I can show myself this by looking in the mirror and moving about. I see my family most days. I can remind myself of this by looking at a recent photo of us together
I can’t speak/no-one is listening to me	I couldn’t speak because I had a tracheostomy/was on a ventilator. I needed it at the time to help me breathe. Even though I couldn’t communicate with them, people were looking after me. Now I can speak and people listen to me. I can prove this by saying this update out loud
I can’t breathe, I’m suffocating	It was difficult for me to breathe because I had pneumonia in my lungs. I was on a ventilator which was helping me breathe. Now, the illness has gone and I can breathe. I can show myself this by taking some deep, slow breaths or exercising
I can’t move, I’m in danger	I couldn’t move because I was in a hospital bed with lots of medical equipment connected to me, and I was on lots of sedating medication. Although I was very ill, I was being given the treatment I needed to save my life. People were looking after me. I can move now. I can show myself this by standing up and moving about freely
I’m on my own, nobody cares	There are lots of staff in ICU but they are very busy and they had lots of people to look after. Visitors weren’t allowed, but I didn’t know that so it makes sense I felt alone. Lots of people care about me. I can remind myself of this by reading all the nice ‘get well’ cards and messages I have had
The doctors and nurses are trying to kill me	I was experiencing delirium which is very common in ICU because of all the medication. People often believe staff are trying to kill them. The things they were doing were part of my medical treatment to keep me alive, but I didn’t know this at the time. They were trying to help me. I can prove this to myself by watching videos of staff in ICU, by returning to the ward and reading the information about emergency tracheotomies the nurse/other expert emailed my therapist
I’m being sexually assaulted	The nurses needed to touch me intimately for medical reasons, like washing me, changing/cleaning my catheter and taking a swab to test for infections (my partner told me this happened at least once). In my confused half-sleep/half-wake medicated state, I didn’t understand why they were touching me then. If someone touches your private parts and you don’t understand why, it makes sense that you might think they are sexually assaulting you, especially because this happened to me in the past. I know now that they weren’t assaulting me. I can remind myself of this by watching the video of the friendly ICU nurse explaining how they care for patients. No-one is touching me now. I have control back over my body and who touches it. I can remind myself of this by looking around to see that there is no-one there, and by gently stroking my skin
I have been abducted	I believed I had been abducted because I woke up in an unfamiliar place and my mind was playing tricks on me because of the drugs I was given. I wasn’t abducted, I was safe in hospital the whole time. I can remind myself of bringing to mind an image of nurses caring for me kindly, even though I wasn’t aware of them at the time
I was in a spaceship/floating down a river/on a journey	Beds in the ICU move to prevent pressure sores. This made me feel like I was moving, and it got mixed up in the dreams I was having. I can remind myself of this by imagining that the spaceship landed safely/I washed safely out of the river/I got home at the end of the journey. I can look around to show myself I am safe in my home now

The latter four examples are common hallucinations and delusions in ICU, but a range of bizarre experiences may be reported. In general, it is useful to update these with normalising information about why such experiences happen, and the knowledge that it did not actually occur. In some cases, patients report confusion about what was real. Usually, logic can help determine whether an experience was likely and some detective work, such as asking friends and family, consulting medical records, or interviewing staff in the ICU can help extrapolate the truth. Often a genuine occurrence has become distorted through delirium. It can be helpful to understand where the hallucination originated from.

Christine believed throughout her time on ICU that she had been abducted by aliens so they could harvest her organs. She had terrifying hallucinations of them cutting her open. Christine and her therapist discussed where this hallucination had come from. It made sense that Christine had believed she had been abducted, as she awoke in a strange place, unable to move. Christine also realised that the staff on ICU had been wearing personal protective equipment, including masks, visors, hair protectors and plastic suits that made them look bizarre and frightening to her. They would have been dressing the surgical wound on her stomach, which might have caused some pain. In her state of being physically ill, and taking lots of painkillers, her mind had interpreted all these experiences as an alien abduction.

#### Trigger discrimination

Careful review of re-experiencing episodes is used to identify memory triggers. Patients are often not aware that their trauma memories are triggered by sensory elements, such as colours, smells, tastes, sounds, being touched on certain parts of the body, body posture, and bodily sensations. Memories may also be triggered by medical settings and reminders such as letters from the hospital, attending appointments, medical TV programmes and media reporting of relevant topics (such as the COVID-19 pandemic). Affect without recollection can initially be hard to spot, but as patients become more aware of their individual triggers, they gradually recognise these emotions as part of their trauma memories.

Once the triggers have been identified, they are intentionally presented, memories/emotional reactions are elicited and then the patient is encouraged to intentionally focus on what is different between ‘then’ (the trauma) and ‘now’ (the reminder). This ‘Then *vs* Now’ discrimination technique can be practised in session and for homework while deliberately introducing the trigger. Noises and pictures similar to those in ICU can be found in internet sound libraries and Google Images. Bodily sensations can be recreated in session. Patients who experienced respiratory distress (e.g. following COVID-19 infection) may feel anxious when they notice breathlessness. For some, this may trigger panic attacks. For patients who only experience panic in response to trauma triggers, this can be treated with ‘Then *vs* Now’ trigger discrimination. For those who have developed panic disorder, where panic attacks occur out of the blue, and are accompanied by catastrophic thoughts, additional cognitive therapy techniques for panic disorder are required.

Krishnan had been admitted to ICU in acute respiratory distress. He was struggling to breathe, and believed he was going to die. Afterwards, Krishnan felt panicky if his breathing was restricted in any way, like if he had anything touching his face (which reminded him of an oxygen mask), or if he got out of breath through exercise. His therapist taught him how to use ‘Then versus Now’ and they practised it in session while holding their hands over their face during and after running on the spot.

#### Site visits

At the time of writing, ICUs are limiting visitors due to the COVID-19 pandemic and site visits are not possible. However, in usual circumstances, ICUs are often happy to facilitate site visits, if they are contacted first. Returning to the ICU is particularly helpful if delirium occurred, to look for updating information (e.g. the nurses are trying to help people, not harm them), and for clues to understand where hallucinations originated. It may also be possible to speak to staff who treated the patient, which can often help fill memory gaps and discover information to update beliefs about what happened.

If you cannot return in person, or as a step before an *in vivo* visit, try a virtual site visit. The outside of the hospital can be revisited using Google Street View, and images of the interior of the hospital can be found online. There are video tours of ICUs available online, such as on the Chelsea and Westminster hospital website (https://www.chelwest.nhs.uk/services/support-services/intensive-care-unit-icu/video-tour).

### Working with meaning: common cognitive themes

As well as using cognitive strategies to address appraisals at the time of the trauma (to include as hotspot updates), we also work on appraisals made since the trauma. Again, these are personal and idiosyncratic to the patient, but certain themes are quite common, as follows.

#### Beliefs about mental illness due to delirium experiences

The term ‘ICU psychosis’ is sometimes used by medics, which can give the impression to patients that they have developed a psychotic illness. Others have specific beliefs or fears about mental illness, which may be exacerbated by PTSD symptoms e.g. ‘the flashbacks mean I have permanently lost my mind’, or may feel ashamed about the way they behaved during their treatment. The psychoeducation we have already described is often helpful to address these beliefs. Additionally, gathering further information through speaking to an expert (such as an ICU staff member), reading about delirium or distributing a survey via patient forums such as on the ICU Steps website can help to address beliefs. We also use behavioural experiments to test beliefs such as ‘I can’t trust my mind’, such as deliberately trying to ‘go crazy’.

#### Beliefs about permanent change

Beliefs related to other types of permanent change are also common e.g. ‘my life will never be the same again’ and ‘I’m not myself anymore’. These are often exacerbated by genuine physical and lifestyle changes and losses that have resulted from the illness or injury, which should be acknowledged and mourned. Often patients are very focused on what has changed, and less aware of what has not, so a useful exercise can be to discuss which aspects of the person and their life have not been lost, or can be reclaimed. For example, superficial physical changes may have occurred, but not fundamental aspects of someone’s identity such as their character and personality, their friends and family and their values. We can also identify and challenge beliefs relating to loss of confidence in one’s own abilities (‘I can no longer be trusted to look after my family’).

Scars and other physical changes can be triggers to trauma memories, exacerbating how severe the changes feel. We use techniques such as photo and video feedback to give a realistic perspective of physical changes, and encourage patients to reduce avoidance of the affected part of their body, such rubbing lotion into their scars every day. Beliefs about how others view their changes in appearance or abilities can be addressed using surveys, including surveys that include photos of the patient’s scars, alongside those of other people, and behavioural experiments in allowing their scars to be visible to others. In our experience, surveys and experiments often show that people show curiosity about scars and other forms of disability, and may look briefly, but do not find them disgusting or repulsive.

#### Health anxiety

Patients may understandably fear becoming unwell again, and experience an increased sense of vulnerability, which may also generalise to fearing for the wellbeing of their loved ones. As with other types of risk appraisal, we use guided discovery, and advice from experts (such as the patient’s medical team) to calculate a realistic probability of becoming critically ill again. This may be somewhat higher than average for patients with ongoing health conditions, but is often not as high as patients fear. Medics can also advise on appropriate precautions to avoid further illness (e.g. living a generally healthy lifestyle), and which precautions may be unnecessary (e.g. constant symptom checking or scanning).

#### Guilt about survival

ICU patients are often aware of others dying on the ward and some report feeling guilty that they have survived when others have not. The high mortality rate associated with COVID-19, including amongst staff, may mean this is a particularly common appraisal amongst these patients. Some patients believe that they were somehow responsible for the death of another patient, such as by taking resources away from them. For others, guilt is linked to general beliefs about equity, for example their survival has broken unwritten rules that the world should be fair, and that things happen for a reason. In treatment, we can address appraisals about responsibility, and beliefs such as ‘I took their place’ or ‘the other person was more worthy of living than me’, and look for alternative explanations for their survival such as ‘the other person was just sicker than me’ and ‘no-one deserved to die, including me’. Surveys can be helpful to gather other opinions, as can work on developing compassion for the self, and reducing rumination.

#### Beliefs about mistreatment and mistrust of healthcare professionals

Some patients report feeling angry about their medical treatment. This ranges from minor concerns about lack of communication or staff availability to claims of clinical negligence or malpractice. Interventions include allowing the person to vent their distress (including writing an anger letter which is not sent), gently probing for any areas of misunderstanding or misappraisals (especially in the case of delirium), reviewing possible reasons for perceived failures (e.g. inattention due to busy wards and long working hours), facilitating communication with the ICU team to address concerns and, if appropriate, helping patients to make a formal complaint. It can also be helpful to consider the advantages and disadvantages of holding on to anger, and helping patients to reduce rumination.

In some cases, patients develop more generalised issues with trusting healthcare professionals. This is important as it can interfere with access to medical treatment, which may be crucial to their ongoing recovery, and can affect the therapeutic relationship. Here it can be helpful to ask the patient to list all the healthcare workers they have ever encountered (usually many) and identify which ones have been untrustworthy or incompetent (usually the minority). Facilitating ways to gather information from healthcare professionals, for example through surveys and online research, can help address beliefs such as ‘they don’t care about their patients’.

### Address maintaining behaviours/cognitive strategies

Patients understandably develop behaviours and cognitive strategies to try to reduce the sense of threat which is central to the experience of PTSD. Following ICU, these commonly include over-protecting others, checking behaviours, internal scanning for symptoms, ruminating and avoidance of reminders such as looking at or touching scars as well as avoidance of activities which are believed to be risky, such as swimming, walking or other physical activity. In therapy, we draw attention to the role these strategies have in maintaining the PTSD, restructure related beliefs (such as those concerning risk), and carry out behavioural experiments (including during therapy sessions) to demonstrate the effects of the behaviours, for example increasing and then decreasing the behaviour (see Maria’s example below). We encourage patients to experiment with dropping their behaviours as homework to test related beliefs. For example, someone who avoids exercise for fear that breathlessness will result in them requiring hospitalisation could experiment with periods of gentle exercise that leads to increased respiration, to test whether they then become seriously ill again. Any concerns about ongoing physical health risks should be checked with the patient’s medical team.

Maria had developed a number of checking behaviours since a medical emergency leading to an admission into ICU. Despite her doctor saying it was unnecessary, Maria took her temperature and her blood pressure every morning. She ruminated about the warning signs she believed she should have noticed before her illness. In therapy, Maria agreed to a behavioural experiment to test the effects of these behaviours on her anxiety. For the first half of the week, she checked her temperature and blood pressure several times a day and ruminated on warning signs. For the second half of the week, she made no checks, and engaged in a reclaiming your life activity every time she noticed herself ruminating. Maria found that her anxiety was slightly higher on the first day that she didn’t do her checks, and her mind kept returning to it during the day. However, by the second day, she felt less anxious, and did not become unwell. She realised that her checking behaviours were fuelling her anxiety and weren’t needed to stay well.

### Some additional considerations

#### Your therapy environment

Be aware that your therapy setting may be triggering for post-ICU patients, especially if you work in a hospital. Introduce ‘Then *vs* Now’ discrimination early on and be prepared to alter aspects of your physical environment and clothes to make the patient more comfortable. Appointments over video conferencing, or telephone sessions, should be offered to patients who cannot attend appointments in person, especially if they have ongoing health problems or disability. If you are working remotely, ask your patient to prepare a suitable home therapy environment. For example, they should keep reminders of the here and now (such as scents that are different from those at the hospital, post-ICU photos of a pleasant day spent with significant others, a sweet or mint for a different taste) nearby during a session, make sure they have peace and quiet for the call, and arrange something pleasant and relaxing to do afterwards.

#### The therapeutic relationship

As health professionals, we ourselves may be a trigger to our patients. Furthermore, beliefs about placing trust in others, and being let down or ignored may affect the therapeutic relationship. It is important to address these early to prevent therapy drop-out and to develop a therapeutic relationship which feels safe, respectful, collaborative and supportive. Another important consideration is to empathise as emphatically with trauma memories that were delusions and hallucinations as you would with ‘real’ traumatic events. These are sometimes bizarre, and patients may have been laughed at or dismissed when they have reported them before. However, they are often quite terrifying and sometimes shameful, and were experienced at the time as completely real. A useful thought exercise is to imagine if the hallucinations had been a real event, they would be some of the most disturbing we have ever encountered.

#### Involving family and partners

It is often valuable to involve family, friends and partners in therapy, and especially following ICU trauma, as social support during and after ICU is a protective factor for PTSD (Deja *et al.*, 2006). Supporters can help in many ways, including filling in memory gaps, helping to explain areas of confusion, encouraging reclaiming your life activities, and accompanying patients on behavioural experiments and site visits, if the therapist cannot. Be aware that PTSD is also common in family members of ICU patients (Jones *et al.*, 2004) and being an informal carer also confers a risk of psychological problems (van den Born-van Zanten *et al.*, 2016). If needed, family members can be directed to treatment themselves, or to sources of other support such as internet forums and support groups.

## Key practice points

(1) A significant minority of patients who have been treated in ICU will develop symptoms of PTSD.
(2) Evidence-based psychological treatment for PTSD, such as CT-PTSD, should be offered to these patients.
(3) All the treatment strategies in CT-PTSD can be used, including work on updating trauma memories, even if there are memory gaps or they include hallucinations or delusions.
(4) Helping patients to make sense of their experiences and addressing beliefs associated with the trauma are important parts of treatment.
(5) Treatment will need to take into account associated physical health difficulties.
(6) Intrusive trauma memories contribute to negative appraisals about how the physical consequences of the trauma appear do others, which can be addressed with video feedback and surveys.
